# Anaphylaxis Induced by Sugammadex in a Patient with Papillary Serous Carcinoma of the Uterine Adnexa Undergoing Exploratory Laparotomy

**DOI:** 10.7759/cureus.3871

**Published:** 2019-01-12

**Authors:** Allan R Escher, Jonathan B Cohen

**Affiliations:** 1 Anesthesiology, H. Lee Moffitt Cancer Center and Research Institute, Tampa, USA

**Keywords:** anaphylaxis, arrhythmia, hypotension, tryptase, iatrogenic anaphylaxis, medication allergy, adverse drug reactions, epinephrine, adrenaline, sugammadex

## Abstract

In this case report, we present a patient who developed anaphylaxis immediately after sugammadex administration. A 67-year-old female with the diagnosis of papillary serous carcinoma was scheduled for an exploratory laparotomy exam under anesthesia and total abdominal hysterectomy. At the end of the operation, sugammadex was administered; it was rapidly accompanied with marked hypotension and bradycardia.

Multiple boluses of phenylephrine were administered with minimal effect. Boluses of epinephrine and vasopressin were given and the patient's hemodynamic instability rapidly abated. A 12-lead electrocardiogram (ECG) obtained in the post-anesthesia care unit (PACU) showed sinus tachycardia and a prolonged corrected QT (QTc) interval. A tryptase level was drawn in the operating room. After an uneventful PACU stay, the patient was observed overnight in the intensive care unit (ICU) as a precaution. Cardiology service was consulted, and they agreed with the anesthesia team that the cause of the patient's hemodynamic instability collapse was consistent with the diagnosis of anaphylaxis. The serum tryptase returned with a level of 62.3 ng/mL, confirming the diagnosis. The patient was discharged on postoperative Day 4.

Anaphylaxis may result from sugammadex usage, and this might cause severe hypotension and cardiac arrhythmias. The prompt recognition and treatment of hypotension and anaphylaxis are critical to minimize morbidity and prevent mortality in these patients.

## Introduction

Synthetic cyclodextrin derivatives, such as sugammadex, show promise as a safe and well-tolerated agent for the reversal of non-depolarizing neuromuscular blocking agents and the prevention of residual blockade [1]. It is the most recent attempt by the pharmaceutical industry to formulate the "ideal reversal agent." Such an agent would have a rapid onset, be able to provide reversal for a range of neuromuscular blockades, be inexpensive, and be safe when used as directed. Sugammadex seems to satisfy most of these characteristics and incorporates a novel mechanism of action. It encapsulates rocuronium and other aminosteroid neuromuscular blocking agents (NMBAs), facilitating the swift reversal of neuromuscular blockade. As opposed to traditional reversal agents, such as neostigmine, sugammadex can also provide reversal from deep blockade [1]. This ability to provide rapid reversal of s neuromuscular blockade without the side-effects of neostigmine has led to its rapid adoption in practice in the United States, European Union, and Japan.

In Japan, it has been in use since 2010; a three-year retrospective study revealed an incidence of sugammadex-induced anaphylaxis at 0.039% (n=6; 95% confidence interval (CI), 0.014%-0.084%), which could be comparable to that of succinylcholine or rocuronium [2]. Following the withdrawal of high-profile medications, such as rofecoxib, aprotinin, and rapacuronium, clinicians have a good reason for caution with adopting new medications into routine clinical practice. In fact, these concerns slowed the introduction of sugammadex in the United States until 2015, when it received approval from the US Food and Drug Administration (FDA) [3]. We report a case of anaphylaxis following sugammadex administration in a patient with papillary serous ovarian carcinoma. The patient had no history of hypersensitivity to sugammadex. In addition, she had received seven cycles of neoadjuvant therapy with carboplatin and paclitaxel prior to surgery.

## Case presentation

A 67-year-old, 115.3 kg, 157-cm female patient with papillary serous adenocarcinoma of the uterine adnexa and uterine masses was brought to the operating room for an exam under anesthesia, exploratory laparotomy, total abdominal hysterectomy, bilateral salpingo-oophorectomy, omentectomy, debulking, and lymph node dissection. Her medical history included hypertension, hemorrhoids, morbid obesity (body mass index (BMI) 46.8), and a pulmonary embolism three months prior to surgery. Enoxaparin sodium was stopped two days prior to surgery. The patient’s functional capacity was moderate to excellent at greater than or equal to four metabolic equivalents (METS). She reported completion of all household chores, cycled three to five miles daily and was capable of climbing two flights of stairs. She reported no history of anesthetic complications and no family history of anesthetic complications. Her surgical history included one previous colonoscopy, for which she received deep sedation.

The patient's home medications included prochlorperazine 10 mg as needed after chemotherapy, dexamethasone 4 mg for three days only after chemotherapy, rivaroxaban 20 mg daily, and enoxaparin 120 mg/0.8 mL solution subcutaneous (held two days prior to surgery). In addition, hydrochlorothiazide-valsartan 12.5-320 mg was taken by the patient daily for control of essential hypertension. A non-smoker, the patient reported one alcoholic beverage an average of four times per month. Her medication allergies consisted of benazepril (tachycardia), ezetimibe (myalgias), simvastatin (myalgias), and triamterene (myalgias).

Premedication for anxiolysis was accomplished with 2 mg midazolam. In the operating room, standard anesthesia monitors were applied, including non-invasive blood pressure measurement, electrocardiogram, peripheral oxygen saturation, and a temperature-sensing Foley catheter. Her baseline vital signs were a temperature of 36.94 degrees Celsius, blood pressure 138/80 mm Hg, heart rate 81 beats/min, and oxygen saturation (SaO_2_) 99%. Induction of general anesthesia proceeded with the administration of 180 mg of 1% propofol, 70 mg lidocaine, and 50 mcg fentanyl after preoxygenation with 100% oxygen. Tracheal intubation was facilitated with the administration of 40 mg rocuronium. A 7.0 mm cuffed oral endotracheal tube was placed with one attempt and then secured at 22 cm. The maintenance of anesthesia with sevoflurane was initiated and the patient received 10 mg of intravenous (IV) dexamethasone after the start of surgery. Mechanical ventilation with volume control was selected with 6 mL/kg and a fraction of inspired oxygen (FiO_2_) of 0.5. A second 18 g IV was started in the left hand and an arterial line was placed in the right radial artery with one attempt.

**Figure 1:**
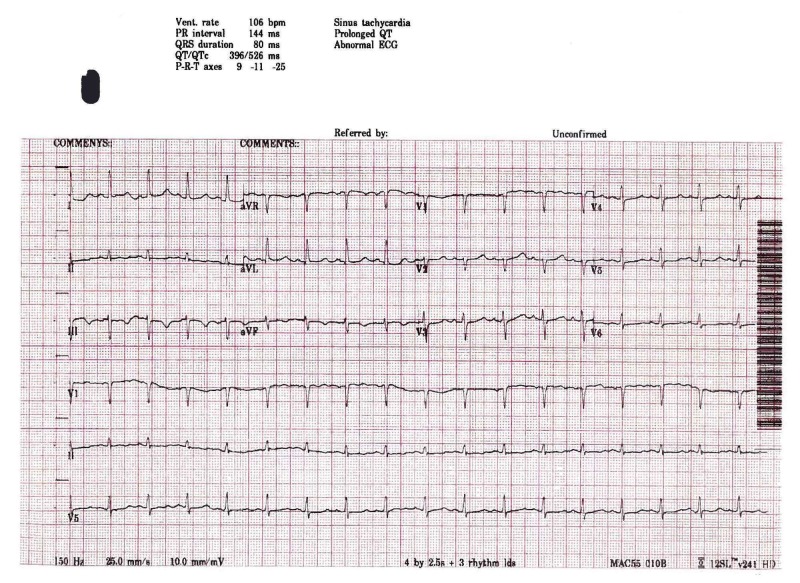
ECG upon admission to PACU ECG: electrocardiogram PACU: post-anesthesia care unit QTc: corrected QT interval QT: time from start of the Q wave to end of the T wave Vent: ventricular PR: time from start of p wave to start of the QRS complex

At the conclusion of the surgery, 400 mg IV sugammadex (3.74 mg/kg) was given as reversal for the neuromuscular blockade (the train-of-four was 0/4). Within 60 seconds, her blood pressure dropped to 58/39 mm Hg, heart rate remained stable at 80, and SpO_2_ dropped to 94%. Phenylephrine was titrated in 0.2 mg doses for a total of 1 mg. Intravenous fluids were administered liberally and the surgical team notified of intractable hypotension. Ischemic changes were observed in electrocardiogram (ECG) leads II and V at the same time that the heart rate began to increase. Given the presentation of symptoms in close proximity to the administration of sugammadex, the possibility of anaphylaxis was considered. At this time, 150 mcg IV epinephrine was given followed an additional dose of 100 mcg. Vasopressin two units IV was then administered to restore the blood pressure to baseline in the setting of epinephrine-induced tachycardia. Auscultation revealed clear breath sounds and there was no increase in airway pressures noted. However, a maculopapular rash was noted over the upper thorax. Greater attention was given to the possibility of anaphylaxis at this point and diphenhydramine 50 mg and famotidine 20 mg were administered. A serum tryptase level was sent to the laboratory to assist with determining the cause of the patient's symptoms. After her condition was stabilized, the patient was successfully extubated and transferred to the recovery room. A 12-lead electrocardiogram showed sinus tachycardia and a prolongation of the corrected QT (QTc) interval (Figure 1).

Cardiology was consulted and the patient was observed in the intensive care unit overnight as a precaution. Dermatological manifestations resolved within a few hours. Serum tryptase from the time of the episode returned at 62 ng/mL (normal < 11.4 ng/mL), which effectively confirms that the cause of this patient's symptoms was consistent with an anaphylactic reaction. She was discharged home on postoperative Day 4 with a recommendation for follow-up with her primary care physician.

## Discussion

The introduction of sugammadex has led to its widespread use in Europe, Japan, and the United States, and it has demonstrated safety in patients with pulmonary disease at the 2 mg/kg and 4 mg/kg dosing regimens [4]. However, a recent study showed hypersensitivity can occur in healthy subjects without a history of prior exposure [3,5]. Data on the epidemiology of perioperative anaphylaxis is variable, with an incidence ranging from 1/1,250 to 1/18,600 per administration [6]. This wide range reflects the difficulty of determining precisely what factors may predispose a patient to hypersensitivity or anaphylaxis. Both in vivo (i.e. skin tests) and in vitro (i.e. basophil activation tests) have demonstrated positive test results with sugammadex [7].

The challenge in the operating room is the myriad of medications that our patients are commonly exposed to, which may result in anaphylaxis: neuromuscular blocking agents (NMBAs), antibiotics, blood products, dyes, and latex [8]. In the case at hand, symptoms manifested within 60 seconds of drug administration. In a prior study, signs of anaphylaxis appeared in 14 out of 15 patients, with a mean time of 1.9 minutes (1 min and 52 s) after a dose of sugammadex [9]. In this case, the placement of an arterial line facilitated the prompt recognition of hypotension.

The rapid administration of epinephrine remains the intervention of first choice because of its alpha-1 agonist vasoconstrictor effects, beta-1 agonist chronotropic and inotropic effects, and beta-2 agonist effects [9]. A 25-year retrospective review of anaphylaxis mortality showed only 23% of individuals received epinephrine before cardiac arrest [10]. Mortality from iatrogenic anaphylaxis improved, with 42% of patients receiving epinephrine prior to cardiac arrest [10]. It is helpful to understand the risk factors that may predispose a patient to anaphylaxis from neuromuscular blocking agents.

A retrospective review of 2,022 cases of NMBA hypersensitivity revealed 1,247 cases of severe NMBA-induced anaphylaxis and 84 fatalities (4.1%) [11]. A multivariate analysis concluded the risk factors associated with a fatal outcome: male gender, emergency surgery, a history of hypertension or another cardiovascular disease, obesity, and ongoing beta‐blocker treatment [11]. Our patient had the following risk factors: hypertension, a history of pulmonary embolus, and obesity.

Serum tryptase is an important marker of mast cell activation; levels may peak after one to two hours and then decline with a t1⁄2 of 1.5 to 2 hours [12]. The specimen should be obtained rapidly in cases of suspected anaphylaxis. Additionally, clear communication with our surgical colleagues should occur when a further neuromuscular blockade is requested, particularly at the end of the case when the risks and benefits can be addressed [13].

## Conclusions

It is important to remember that perioperative anaphylaxis can occur in healthy patients without a prior history of hypersensitivity to NMBAs. The prompt recognition of perioperative anaphylaxis is critical since symptoms may present suddenly. Epinephrine remains the first-line treatment for perioperative anaphylaxis, to reduce morbidity and mortality. Additionally, the clinician should be aware of the risk factors for mortality from the use of NMBAs: male gender, emergency surgery, hypertension or cardiovascular disease, obesity, and ongoing beta-blocker therapy. Serum tryptase levels should be obtained in order to help clarify the clinical picture, although the results will not be immediately available at the point of care. Finally, the re-administration of neuromuscular blocking agents should take place with interdisciplinary communication and collaboration, especially when re-dosing occurs at the end of a case.
